# Short tandem repeat profiling for the authentication of cancer stem‐like cells

**DOI:** 10.1002/ijc.33370

**Published:** 2020-11-09

**Authors:** Paola Visconti, Federica Parodi, Barbara Parodi, Lucia Casarino, Paolo Romano, Mariachiara Buccarelli, Roberto Pallini, Quintino Giorgio D'Alessandris, Andrea Montori, Emanuela Pilozzi, Lucia Ricci‐Vitiani

**Affiliations:** ^1^ IRCCS Ospedale Policlinico San Martino, Interlab Cell Line Collection (ICLC), Biological Resource Center (CRB‐HSM) Genoa Italy; ^2^ Department of Legal and Forensic Medicine University of Genoa Genoa Italy; ^3^ IRCCS Ospedale Policlinico San Martino, Proteomics Service, Scientific Direction Genoa Italy; ^4^ Department of Oncology and Molecular Medicine Istituto Superiore di Sanità Rome Italy; ^5^ Fondazione Policlinico Universitario A. Gemelli IRCCS ‐ Institute of Neurosurgery, Catholic University School of Medicine Rome Italy; ^6^ Department of Clinical and Molecular Medicine, Sant'Andrea Hospital University "La Sapienza" Rome Italy

**Keywords:** cell line authentication, colorectal tumor, glioblastoma, human stem‐like cell lines, short tandem repeat profiling

## Abstract

Colorectal and glioblastoma cancer stem‐like cells (CSCs) are essential for translational research. Cell line authentication by short tandem repeat (STR) profiling ensures reproducibility of results in oncology research. This technique enables to identify mislabeling or cross‐contamination of cell lines. In our study, we provide a reference dataset for a panel of colorectal and glioblastoma CSCs that allows authentication. Each cell line was entered into the cell Line Integrated Molecular Authentication database 2.1 to be compared to the STR profiles of 4485 tumor cell lines. This article also provides clinical data of patients from whom CSCs arose and data on the parent tumor stage and mutations. STR profiles and information of our CSCs are also available in the Cellosaurus database (ExPASy) as identified by unique research resource identifier codes.

## What's new?

Human cell lines obtained from cancer stem‐like cells represent an invaluable model for studying tumor properties. Cell line authentication by short tandem repeat (STR) profiling is an important tool to identify the potential mislabeling or cross‐contamination of cell lines. Here, the authors characterized 18 colorectal cancer stem‐like cell lines from 17 patients and 103 glioblastoma cancer stem‐like cell lines from 95 patients by STR profiling to create a reference dataset that allows the authentication of these cell lines and their identification through a unique research resource identifier. The results will help further ensure the reliability and reproducibility of research experiments.

**Abbreviations**
AMELX: amelogenin in Xp22.1‐22.3
AMELY: amelogenin in Yp11.2
CLASTR: cellosaurus STR database
CLIMA: Cell Line Integrated Molecular Authentication database
CSC: cancer stem‐like cell
CTSC: colorectal tumor stem‐like cell
GSC: glioblastoma stem‐like cell
ICLAC: International Cell Line Authentication Committee
ICLC: Cell Bank Interlab Cell Line Collection
MGMT: O6‐methylguanine DNA methyltransferase
MSI: microsatellite instability
PBMC: peripheral blood mononuclear cell
PCR: polymerase chain reaction
STR: short tandem repeat

## 1 INTRODUCTION

Cancer research requires models that use patient‐derived cultured cells. These models allow to study tumor heterogeneity, in particular in the early stages of tumorigenesis. Isolation and in vitro cultivation of cancer stem‐like cells (CSCs), a small fraction of self‐renewing cells with stem‐like properties, provide a model to study the properties of these tumor initiating cells.^1^, ^2^, ^3^, ^4^, ^5^, ^6^ Stem‐like cells are grown as free‐floating oncospheres in serum‐free medium supplemented with growth factors.^1^, ^2^, ^3^, ^4^, ^5^, ^6^ When grafted as orthotopic models, CSCs closely reproduce the parent tumor, both histologically and genetically. Therefore, the identification of each cell line is essential in oncology research.^7^, ^8^, ^9^ Short tandem repeat (STR), a standard authentication technique that allows identification of the individual from whom each cell line originates,^10^ amplifies a set of polymorphic STR markers and then separates the polymerase chain reaction (PCR) products by capillary electrophoresis size fractionation. We characterized 18 colorectal CSC lines (CTSCs)^11^ from 17 patients and 103 glioblastoma CSCs (GSCs)^3^ from 95 patients by STR assay to create a reference dataset that allows the determination of the authenticity of these cell lines and ensures the reliability and reproducibility of research experiments. In one patient with a colorectal tumor and in seven patients with glioblastoma, we established CSC lines from different portions of the same tumor. Moreover, in two glioblastoma patients, of whom we obtained GSC lines from both primary surgery and surgery for recurrence, the STR profile confirmed the authenticity of CSC lines derived from the same patient. Peripheral blood mononuclear cells (PBMCs) and primary tumor cells of some patients were also analyzed. Due to the availability of the surgical samples, we could preserve tumor tissue for genotyping only in a minority of patients.

STR profiling was carried out using standardized procedures for unambiguous authentication and identification of human cell lines according to the American National Standards Institute/American Type Culture Collection Standard ASN‐0002‐2011.^12^ Each cell line profile presented in this artcle was entered in a specific data set (ICLC 3) of the Cell Line Integrated Molecular Authentication database (CLIMA),^13^ including STR profiles obtained in different cell banks by using different platforms. All STR profiles were also compared using the cellosaurus STR database (CLASTR) search tool of the Cellosaurus database (ExPASy) (https://web.expasy.org/cellosaurus/).

The cell line profiles were challenged against public databases to exclude cross‐contamination with commercially available cell lines. Comparison of the cell line profiles against each other ruled out duplicates due to cross‐contamination. Duplicates were found only in those cell lines that derived from different regions of the same tumor.

## 2 MATERIALS AND METHODS

Further method descriptions are included in Supplementary Material and Methods.

### 2.1 CSC cultures

CTSCs and GSCs were isolated from tumor samples through mechanical dissociation and cultured in a serum‐free medium supplemented with growth factors, as previously described.^1^, ^2^, ^3^, ^4^, ^5^, ^6^ Under stem cell culture conditions, proliferating CSC lines actively required 3 to 4 weeks to be established (Supplementary Material and Methods).

### 2.2 Mycoplasma statement

All experiments were performed with mycoplasma‐free cells. Mycoplasma contamination in cell cultures was evaluated using MycoAlert Mycoplasma Detection Kit (Lonza Walkersville Inc, Walkersville, MD).

### 2.3 Molecular analyses in CTSCs and colorectal tumors

Single‐point mutations and small insertions/deletions in CTSCs were assessed by targeted DNA resequencing, focusing on 17 genes known to be frequently mutated in colon cancer, as previously described.^11^

Microsatellite instability (MSI) detection was performed using the Promega panel of mononucleotide MSI markers (MSI Analysis System, Version 1.2, Promega Corporation, Fitchburg, WI). The expression of four mismatch repair proteins, MLH1, MSH2, MSH6 and PMS2, was investigated in tumor tissues corresponding to CTSCs that showed MSI‐High.

The expression of the stem cell marker CD133 and the epithelial antigen Ber‐EP4 in CTSCs was evaluated by flow cytometry, as previously described.^5^

### 2.4 Molecular analyses in GSCs and glioblastoma tissues

Tumor proliferation index was analyzed by immunohistochemistry on paraffin sections of glioblastoma samples using the avidin‐biotin‐peroxidase complex methods (ABC‐Elite kit, Vector, Burlingame, CA).^14^ The anti‐Ki‐67 monoclonal antibody (MIB‐1, Dako) was used. The expression of CD133 and Sox2 in GSCs was evaluated by flow cytometry. O6‐methylguanine DNA methyltransferase (MGMT) promoter methylation patterns were studied by methylation‐specific PCR on genomic DNA.^15^ DNA from normal lymphocytes treated with SssI methyltransferase (New England Biolabs, Ipswich, MA) was used as positive control. PCR products were separated onto 3% agarose gel, stained with ethidium bromide and visualized under UV illumination.

### 2.5 STR profiling

All human cell lines described have been authenticated by STR profiling within the last 3 years. Genomic DNA was isolated from cell pellets using the DNeasy Blood & Tissue Kit (Qiagen, Milan, Italy) and treated with RNase, according to the manufacturer's instructions. Yield was measured with NanoDrop 1000 spectrophotometer (Thermo Fisher Scientific, Wilmington, DE). One nanogram of DNA of each sample was used for the STR analysis. Samples were amplified and electrophoretically separated on an ABI Prism 3100 Genetic Analyzer (Applied Biosystems).

STR profiles were analyzed by the GeneMapper ID software (Applied Biosystems), Version 3.2. The results showed highly reproducible cell line‐specific numeric patterns. The assay was performed by the Cell Bank Interlab Cell Line Collection (ICLC) of the Biological Resource Centre, IRCCS Ospedale Policlinico San Martino of Genoa, in collaboration with the Department of Legal and Forensic Medicine of Genoa University. Comparison of STR profiles using the CLIMA database was performed using the identification feature in CLIMA 2.1 version of the database, as previously described.^13^ Each new cell line profile was compared to profiles of all cell lines (4485 cell lines names and 5587 distinct authentication assays) contained in the database, that are divided into datasets (Table S1). All STR profiles of CTSCs and GSCs were compared using CLASTR, the Cellosaurus STR similarity search tool that contains more than 6400 distinct cell lines with an associated STR profile.^16^

## 3 RESULTS

### 3.1 Patient clinical data and molecular cell line characterization

We characterized 18 CTSCs from 17 patients (7 males and 10 females, mean age 68.4 years). Tumor site and disease stage (grade, pTNM and Dukes) of CTSCs are shown in Table 1. Mutations were harbored by CTSCs in the following genes: *ACVR1B*, *AMER1*, *APC*, *BRAF*, *CTNNB1*, *FBXW7*, *KIAA1804*, *KRAS*, *MAP2K4*, *NRAS*, *PIK3CA*, *PTEN*, *SMAD2*, *SMAD4*, *SOX9*, *TCF7L2* and *TP53* (Table 1; Table S2).

**TABLE 1:** STR profiles, disease stage and principal mutations of CTSCs

Patient ID	Sex	Age	Tumor site	Disease grade	Disease pTNM	Disease dukes	Cell line name	Cellosaurus accession number	ACVR1B	AMER1	APC	BRAF	CTNNB1	FBXW7	KIAA1804	KRAS	MAP2K4	NRAS	PIK3CA	PTEN	SMAD2	SMAD4	SOX9	TCF7L2	TP53	STR ID	AM	D5S818	D13S317	D7S820	D16S539	VWA	TH01	TPOX	CSF1PO	MSI Status	Ref.
1	M	68	Left	G3	IIIB	C	CTSC#1.1	CVCL_A9WH			■							■	■			■		■		254	X,Y	11,13	8,9	10	9,11	16,17	8,9	8	12	MSS	5, 11
1	M	68	Left	G2	IIIB	C	CTSC#1.2	CVCL_A9WI			■		■					■	■			■		■		250	X,Y	11,13	8,9	10	9,11	16,17	8,9	8	12	MSS	5, 11
2	F	66	Right	G2	IIA	B	CTSC#18	CVCL_A9WJ	■		■			■	■	■				■			■	■	■	248	X	11,12	11,12	10,13	11	17	6,9.3	8,9	9,14	MSI‐H	5, 11
3	F	63	Right	G2	IVA	D	CTSC#CRO	CVCL_A9WK																		251	X	11,12	11,14	10,11	9,11	17,19	7,9.3	8	10	MSS	11
4	M	64	Right	G2	IIA	B	CTSC#85	CVCL_A9WL			■					■				■		■				249	X,Y	9,12	8,12	10	11,13	14	6,7	8	10,12	MSS	^11^
5	M	80	Left	G3	IIIC	C	CTSC#383	CVCL_A9WM			■		■			■					■				■	413	X,Y	12	9,11	11,12	12,15	17,18	9,9.3	9,11	10,11	MSS	^11^
6	M	76	Right	G2	IIIC	C	CTSC#389	CVCL_A9WN						■		■			■			■	■		■	500	X,Y	12,13,15	14,15	8,11	9,14	15,18,19	9,9.3	10,12	10,12	MSI‐H	^11^
7	M	57	Right	G3	IIIC	C	CTSC#393	CVCL_A9WT			■		■	■					■							416	X,Y	12	9,12	12	9	18	7,9	8,11	13	MSS	^6^, ^11^
8	F	46	Left	G2	IIIB	C	CTSC#398	CVCL_A9WQ			■					■			■			■			■	415	X	12	8,10	8,10	9,14	14,15	6	10,12	11	MSS	^6^, ^11^
9	M	82	Right	G3	IIIC	C	CTSC#416	CVCL_A9WR	■	■	■	■	■		■		■	■	■	■	■	■	■	■	■	501	X,Y	11,14	7,12	12,13	11,14	15,18	6,9.3	7	11,12,13	MSI‐H	^6^, ^11^
10	F	70	Right	G2	IIA	B	CTSC#417	CVCL_A9WS			■								■						■	417	X	11	11	11	12	16,17	6,8	9	12	MSI‐L	^11^
11	F	68	Right	G3	IIIB	C	CTSC#430	CVCL_A9WT			■			■	■				■			■				498	X	11,15	8,11	8,14	11	16,19,20	9,9.3	9,11	11	MSI‐H	^6^, ^11^
12	M	49	Left	G3	IIIB	C	CTSC#432	CVCL_A9WU					■			■								■	■	499	X,Y	10,13	10,12	10,12	13	17,18	8,9.3	8	10,11	MSS	^6^, ^11^
13	F	87	Right	G3	IVB	—	CTSC#446	CVCL_A9WW			■				■				■				■	■	■	502	X	13	9,12	10,11	11,13	17	9.3	8,11	11	MSS	
14	F	73	Right	G3	IIA	B	CTSC#510	CVCL_A9WY		■	■		■		■	■						■	■	■	■	606	X	12,13	9,12	10,11	9,12	18,2	8,9	8,9	10,11	MSS	
15	F	85	Right	G3	IIIC	C	CTSC#438	CVCL_A9WV		■		■				■			■		■		■	■		579	X	11,14	9,12	9,10	9,10	16,17	6,9	8	11	MSI‐H	^11^
16	F	63	Right	G3	IIIC	C	CTSC#482	CVCL_A9WX		■	■	■		■	■	■			■	■			■	■	■	708	X	9,10	11,12	9,10	10	15,16,17,18	6,9	8,9,10	12,13	MSI‐H	
17	F	66	Right	G3	IIIB	C	CTSC#553	CVCL_A9WZ	—	—	—	—	—	—	—	—	—	—	—	—	—	—	—	—	—	604	X	11,12	11,12	11,12	9,12	14,17	9,10	8	12	MSS	

*Notes:* STR profiles generated in our study from CTSCs. Tumor site and disease stage (grade, pTNM and dukes) of colorectal tumors that originated CTSCs and the principal mutations harbored by CTSCs are also shown. Mutation in the following genes were analyzed: ACVR1B, AMER1, APC, BRAF, CTNNB1, FBXW7, KIAA1804, KRAS, MAP2K4, NRAS, PIK3CA, PTEN, SMAD2, SMAD4, SOX9, TCF7L2, TP53 (details are shown in Table S2). Shown is also MSI status of CTSCs. MSI‐H, MSI‐high; MSI‐L, MSI‐low; MSS, microsatellite stable (details are shown in Table S3). Previous studies involving CTSCs are indicated by the number of reference in the last column of the table.

Abbreviations: F, female; M, male; STR, short tandem repeat; CTSC, colorectal tumor stem‐like cell.

Analysis of MSI status using mononucleotide MSI markers showed that 6 CTSC lines had MSI‐High (CTSC#18, CTSC#389, CTSC#416, CTSC#430, CTSC#438, CTSC#482), one line had MSI‐Low (CTSC#417) and the other lines were microsatellite stable (Table S3). In accordance, expression analysis of the four mismatch repair proteins, MLH1, MSH2, MSH6 and PMS2, assessed in tumor tissues corresponding to the microsatellite instable CTSCs, also showed MSI‐High (Table S3).

CTSCs were analyzed for the expression of CD133 and epithelial antigen Ber‐EP4 (Table S4). CD133 is one of the key stem cell markers for colorectal cancer^5^ and its expression is associated with cell differentiation and tumor size.^17^ In our collection of CTSCs, CD133 and Ber‐EP4 expression was 63.4% ± 6.4% (mean ± SEM; range 23.2%‐99.3%) and 94.8% ± 1.3% (mean ± SEM; range 82.8%‐100%), respectively.

We characterized 103 GSCs from 95 glioblastoma patients (66 males and 29 females, mean age 62.3 years). Tumor location, disease stage (primary/recurrent tumor), *MGMT* gene methylation status and proliferation index (Ki67) of parent tumors are shown in Table 2. *MGMT* gene promoter status was methylated in 46 tumors (49%), unmethylated in 44 tumors (46%) and not available in 5 tumors (5%).

**TABLE 2:** STR profiles, disease stage, MGMT methylation and proliferation index Ki67 (%) of GSCs

Patient ID	Sex	Age	Tumor location	Type of tumor	Cell line name	Cellosaurus accession number CVCL	MGMT	Ki67 (%)	STR ID	AM	D5S818	D13S317	D7S820	D16S539	VWA	TH01	TPOX	CSF1PO	Ref.
19	m	40	Temporal	pt	GSC#1	CVCL_A9T7	M	20	418	X	12,13	8	8,10	11,14	16,18	6,9.3	8	10	^1^, ^2^, ^3^, ^4^
20	m	57	Parietal	pt	GSC#10	CVCL_A9T8	M	30	631	X,Y	11	11,12	10,11	11,12	16,17	9.3	8	11,12	^1^, ^2^, ^3^, ^4^
21	m	77	Parietal	pt	GSC#23C	CVCL_A9T9	UM	50	419	X,Y	11	12	10,11	9,11	17	7	8,9	11,12	^1^, ^2^, ^3^, ^4^
21	m	77	Parietal	pt	GSC#23P	CVCL_A9TA	UM	50	492	X,Y	11	12	10,11	9,11	17	7	8,9	11	^1^, ^2^, ^3^, ^4^
22	m	72	Frontal	pt	GSC#28	CVCL_A9TB	M	5	427	X,Y	10,12	10,11	9,10	10,14	14,16	9,9.3	8,11	10,11	^1^, ^2^, ^3^, ^4^
23	m	44	Frontal	pt	GSC#30P	CVCL_A9TC	M	10	428	X,Y	11,12	12	10,12	12	14,16	6,9	8,11	11,12	^1^, ^2^, ^3^, ^4^
23	m	44	Frontal	pt	GSC#30pt	CVCL_A9TD	M	10	493	X	11,12	12	10	12	14,16	6,9	8,11	11,12	^1^, ^2^, ^3^, ^4^
24	m	59	Occipital	pt	GSC#61	CVCL_A9TE	UM	35	420	X,Y	11	8,12	9,10	10,13	17	9.3	11	11	^1^, ^2^, ^3^, ^4^
25	m	64	Frontal	rt	GSC#62	CVCL_A9TF	M	10	421	X,Y	9	11,12	7,11	12	15,17	6,8	8	10,13	^1^, ^2^, ^3^, ^4^
26	m	48	Parietal	pt	GSC#67	CVCL_A9TG	UM	20	422	X,Y	10,13	11,13	8	9,11	18	6,7	11	10,12	^1^, ^2^, ^3^, ^4^
27	m	58	Parietal	pt	GSC#68	CVCL_A9TH	UM	10	423	X,Y	11,12	10,11	10,11	11,13	15,17	6,9.3	8	10	^1^, ^2^, ^3^, ^4^
28	f	67	Parietal	pt	GSC#70	CVCL_A9TI	UM	20	424	X	11,12	12,13	8,10	11,12	14,17	9	8,11	11	^1^, ^2^, ^3^, ^4^
29	f	70	Frontal	pt	GSC#74	CVCL_A9TJ	UM	15	425	X	11,12	8,12	9	11,12	15	9,9.3	8	10	^1^, ^2^, ^3^, ^4^
30	f	48	Frontal	pt	GSC#76	CVCL_A9TK	UM	15	426	X	10	12	8,12	12	17	7	11	12	^1^, ^2^, ^3^, ^4^
31	m	52	Temporal	pt	GSC#83	CVCL_A9TL	UM	40	445	X	11	12	12	10,11	17,18	6	8,11	10,11	^1^, ^2^, ^3^, ^4^
31	m	52	Temporal	pt	GSC#83.2	CVCL_A9TM	UM	40	446	X	11	12	12,13	10,11	17,18	6	8,11	10,11	^1^, ^2^, ^3^, ^4^
32	f	49	Parietal	pt	GSC#112	CVCL_A9TN	M	18	619	X	12	12	11	11	17	7	11	10	^1^, ^2^, ^3^
33	m	53	Parietal	rt	GSC#120	CVCL_A9TP	UM	30	432	X,Y	11	11	10,11	9,11	14,21	9.3	8	10,13	^1^, ^2^, ^3^
34	m	57	Temporal	pt	GSC#142	CVCL_A9TQ	M	40	429	X	10.13	9	8,10	12	16,17	6,9.3	8,11	11	^1^, ^2^, ^3^
35	f	69	Frontal	pt	GSC#147	CVCL_A9TR	UM	25	440	X	12,13	11	8,12	9,12	15,17	6,8	8	11,12	^1^, ^2^, ^3^
36	m	55	Parietal	rt	GSC#148	CVCL_A9TS	UM	70	433	X	9	10	8,10	12,13	18	6,9.3	8,9	9	^1^, ^2^, ^3^
37	m	69	Occipital	pt	GSC#151	CVCL_A9TT	M	30	434	X,Y	13	12	9,11	11,12	16,18	7,9	11	12,13	^1^, ^2^, ^3^
38	m	56	Parietal	pt	GSC#163	CVCL_A9TU	UM	15	442	X,Y	11,12	12	9,11	11	18	7,9	8	10,12	^1^, ^2^, ^3^
39	m	61	Temporal	pt	GSC#169	CVCL_A9TV	UM	40	436	X,Y	11,12	9	10,11	11,13	14	10	11	10,11	^1^, ^2^, ^3^
40	m	58	Temporal	—	GSC#170	CVCL_A9TW	M	20	643	X	11,12	11	9,12	9,11	14,16	6,9.3	11,12	11,12	^3^
41	m	74	Frontal	rt	GSC#171	CVCL_A9TX	M	10	437	X,Y	12,13	11,13	8,10	9,12	16,17	8,9.3	10,11	10,11	
42	f	77	Parietal	pt	GSC#172	CVCL_A9TY	UM	20	443	X	11	9	9,12	11,13	14,16	7	9,10	11,12	^1^, ^2^, ^3^
43	f	64	Occipital	rt	GSC#181	CVCL_A9TZ	M	10	438	X	11,12	12	11,12	8,9	17,18	9	8	10	
44	f	53	Frontal	pt	GSC#184	CVCL_A9U0	UM	25	441	X	11	12,13	9,10	11	17,18	6,9	8	10,12	^3^
45	m	70	Temporal	pt	GSC#188	CVCL_A9U1	UM	20	439	X,Y	12,13	12,14	10	9,13	18,19	9.3	8,11	11,13	^3^
46	m	73	Frontal	pt	GSC#191	CVCL_A9U2	M	30	444	X,Y	11,13	12	10,11	11	16,17	6,9.3	10,11	11	^3^
47	m	70	Parietal	pt	GSC#195	CVCL_A9U3	UM	40	431	X,Y	12	8,11	7,13	12	14,17	9,9.3	8	11	^3^
47	m	70	Parietal	pt	GSC#195V	CVCL_A9U4	UM	40	447	X,Y	12	8,11	7,13	12	14,17	9,9.3	8	11	
48	f	71	Parietal	pt	GSC#196	CVCL_A9U5	M	25	775	X	12,13	11,12	10,11	10,11	17	7,9	10, 11	11	^1^, ^2^, ^3^
49	f	80	Parietal	pt	GSC#204	CVCL_A9U6	M	25	473	X	10,12	12	11,12	9,12	17,18	6,9.3	8,9	9,11	^1^, ^2^, ^3^
50	m	76	Temporal	pt	GSC#206	CVCL_A9U7	M	50	474	X	10,13	11,13	9,12	11,12	16	9.3	8,10	9,12	^3^
51	m	68	Temporal	rt	GSC#208	CVCL_A9U8	UM	40	475	X,Y	11	11	10,11	12,13	15,16	9,9.3	8	11	^1^, ^2^, ^3^
52	f	43	Occipital	pt	GSC#209	CVCL_A9U9	M	40	507	X	11,13	11	10,13	12	13,16	9	11	10,11	^1^, ^2^, ^3^
53	m	53	Parietal	pt	GSC#210	CVCL_A9UA	UM	40	476	X,Y	12	11,12	8,12	11,12	16,18	8,9	8,11	9	^1^, ^2^, ^3^
54	m	49	Frontal	rt	GSC#213	CVCL_A9UB	M	30	477	X,Y	10,11	8,12	8,11	10,11	17,18	6,8	8	11,12	^1^, ^2^, ^3^
55	m	62	Frontal	pt	GSC#220C	CVCL_A9UC	UM	30	478	X,Y	11,13	11,13	8,11	10,11	14,17	7,9	8	12,13	^1^, ^2^, ^3^
56	m	77	Temporal	pt	GSC#221	CVCL_A9UD	UM	40	479	X,Y	11,13	8,11	8,13	11,12	16,17	9,9.3	9,11	10	^1^, ^2^, ^3^
57	m	64	Temporal	pt	GSC#242	CVCL_A9UE	M	25	480	X,Y	12	11	10,14	9,11	17,18	8	8,11	11,12	^1^, ^2^, ^3^
58	m	57	Multicentric	pt	GSC#257	CVCL_A9UF	M	50	481	X,Y	12,14	12	9,10	11,12	14,18	8,9.3	8,11	12,13	^1^, ^2^, ^3^
59	m	38	Temporal	pt	GSC#262	CVCL_A9UG	UM	35	482	X,Y	11	11	10,11	12,13	16,18	6,9	8	12	^1^, ^2^, ^3^
60	m	58	Occipital	pt	GSC#275	CVCL_A9UH	M	50	483	X,Y	10,12	10,12	8,12	9,13	16	6,10	8	10,11	^1^, ^2^, ^3^
60	m	58	Occipital	rt	GSC#275bis	CVCL_A9UI	M	50	484	X,Y	10,12	10,12	8,12	9,13	16	6,10	8	10,11	
61	m	56	Temporal	pt	GSC#277	CVCL_A9UJ	UM	NA	508	X,Y	12	11	8,10	12,13	16,17	6,8	8,11	10,12	^3^
62	m	60	Frontal	pt	GSC#284	CVCL_A9UK	UM	NA	485	X,Y	11	11	10	9,11	14	8,9	8	11,13	^1^, ^2^
63	m	50	Cerebellar	pt	GSC#290	CVCL_A9UL	UM	NA	486	X,Y	11	11	9,13	10,13	15,19	7,9	8,9	11,12	
64	f	61	Frontal	pt	GSC#291	CVCL_A9UM	M	40	487	X	12	8,14	10,14	11,12	17,19	7,9	8,10	12	
65	m	47	Frontal	pt	GSC#298	CVCL_A9UN	UM	30	488	X,Y	11,13	9,11	7,11	11,12	15,17	8,9	8	10,11	
66	m	65	Temporal	pt	GSC#309S	CVCL_A9UP	M	5	489	X	11,13	8,12	9,12	14	16	9,9.3	8	11,12	
67	f	54	Parietal	pt	GSC#314C	CVCL_A9UQ	M	40	491	X	12,13	11,14	10,12	11	16,19	6,9.3	8,11	10	
67	f	54	Parietal	pt	GSC#314P	CVCL_A9UR	M	40	490	X	12,13	11,14	10,12	11	16,19	6,9.3	8,11	10	
68	m	53	Frontal	pt	GSC#315	CVCL_A9US	UM	50	629	X,Y	9	9,12	10,12	12	16,18	9,9.3	9,10	10	
69	m	69	Temporal	pt	GSC#318	CVCL_A9UT	M	NA	509	X,Y	12	11,12	9,10	11,12	16,19	8,9.3	9,11	10,12	
70	f	51	Parietal	pt	GSC#323	CVCL_A9UU	NA	40	644	X	11,12	11,12	11	11,13	18	8,9.3	11	12,13	
71	m	70	Temporal	pt	GSC#326	CVCL_A9UV	UM	45	645	X,Y	9,11	10,12	8,12	11,12	16,17	7,9.3	8,9	12	
72	m	82	Frontal	pt	GSC#327	CVCL_A9UW	UM	60	630	X,Y	13	11	8,10	11	17,18	9,9.3	8	10,11	
73	m	73	Occipital	pt	GSC#329	CVCL_A9UX	M	30	510	X,Y	11,13	8,14	10,11	9,10	17,19	6	8	11,12	
74	m	70	Temporal	pt	GSC#352	CVCL_A9UY	UM	40	657	X,Y	11,13	12,13	9,11	8,11	16,17	6,7	9	11	
75	m	61	Frontal	pt	GSC#361	CVCL_A9UZ	UM	50	658	X,Y	11,12	8,12	10	13	14,17	6,7	8,9	10,12	
76	m	54	Temporal	pt	GSC#365	CVCL_A9V0	UM	50	656	X,Y	9,12	11	8,10	10,11	18	6,8	8	11	
77	m	43	Temporal	pt	GSC#366	CVCL_A9V1	UM	35	652	X,Y	11	11,12	8,9	10,13	16,17	9	8,11	12	
78	f	65	Frontal	pt	GSC#369	CVCL_A9V2	M	40	660	X	13	11,12	10	11,12	15	6,9.3	8	10	
79	m	69	Frontal	pt	GSC#381	CVCL_A9V3	M	30	653	X	11,13	11	10,11	11,13	14,17	9	8	11	
80	f	54	Temporal	pt	GSC#384	CVCL_A9V4	M	50	632	X	11,12	12	10,11	10	16,17	7,9	8,11	10,12	
81	m	72	Occipital	pt	GSC#389	CVCL_A9V5	UM	30	655	X,Y	12	8,11	9,10	11,12	14,17	6	8	10,11	
82	f	62	Temporal	pt	GSC#391	CVCL_A9V6	M	15	723	X	10,13	12	8,12	12	16	9,9.3	9,10	9,10	
83	m	70	Temporal	pt	GSC#393	CVCL_A9V7	UM	25	724	X	11	8,14	10	12,13	17,18	9,10	8	10	
84	m	64	Frontal	pt	GSC#394	CVCL_A9V8	M	30	665	X	13	12,13	10,11	11,13	16,17	9.3,10	9,11	11,12	
84	m	64	Frontal	rt	GSC#394bis	CVCL_A9V9	M	10	670	X,Y	13	12	10,11	11,13	16,17	9.3,10	9,11	11,12	
85	m	49	Temporal	pt	GSC#395	CVCL_A9VA	M	25	627	X	9,11	12	8,11	13	16,17	6,9.3	8,11	10,11	
86	f	62	Parietal	pt	GSC#397	CVCL_A9VB	NA	25	654	X	9,11	11,12	9,11	11	15,18	9.3	8,11	10,11	
87	m	67	Temporal	pt	GSC#399	CVCL_A9VC	NA	20	623	X,Y	12	12	8,12	11,12	16,17	7,9.3	11	11,12	
88	m	71	Occipital	pt	GSC#401	CVCL_A9VD	M	25	633	X,Y	11,12	13	10,11	11,13	15	6,9.3	8	11,12	
89	f	68	Frontal	pt	GSC#403	CVCL_A9VE	M	25	624	X	9,11	11,12	12	11	14,15	7,9.3	9,11	12,13	
90	m	66	Temporal	pt	GSC#406	CVCL_A9VF	M	20	628	X,Y	12,13	14	10,12	9,12	15,16	9.3	8,9	10	
91	f	70	Frontal	pt	GSC#407	CVCL_A9VG	NA	20	651	X	12	9	10,11	11,13	19	9	11	10,11	
92	m	69	Temporal	pt	GSC#411	CVCL_A9VH	M	20	646	X	8,11	9,11	8,11	11,12	16,19	9.3	8	10,12	
93	f	62	Parietal	pt	GSC#413	CVCL_A9VI	NA	20	625	X	12,13	12	10	11,14	16,17	8,9.3	8,11	10	
94	m	71	Frontal	pt	GSC#415	CVCL_A9VJ	UM	15	634	X,Y	12	11,13	8	8,11	18	9,10	8,10	11,12	
95	f	85	Frontal	pt	GSC#416	CVCL_A9VK	NA	20	648	X	10	11	8,10	12,13	14,18	9,9.3	8,11	10	
96	f	52	Frontal	pt	GSC#420	CVCL_A9VL	M	25	635	X	11,13	12	8,11	12	15,17	6,7	10	11	
97	m	—	Frontal	pt	GSC#421	CVCL_A9VM	UM	20	663	X,Y	10,12	11	9,11	10,12	16,17	9	8,12	10,12	
98	f	70	Temporal	pt	GSC#426	CVCL_A9VN	UM	25	668	X	12,13	8,12	10,(13)	9	18	6,9	11	10,11	
99	f	—	Temporal	pt	GSC#429	CVCL_A9VP	UM	20	722	X	13,15	12,13	9,10	9,12	15,18	8	8,11	9,11	
100	m	53	Occipital	pt	GSC#431	CVCL_A9VQ	M	40	671	X,Y	13	11	8,11	8,10	17,18	6	9,10	10	
101	m	67	Parietal	pt	GSC#432	CVCL_A9VR	M	NA	700	X,Y	12,13	8,12	12,13	11,12	16	8	8	10,12	
102	f	57	Temporal	pt	GSC#433	CVCL_A9VS	M	20	666	X	11,13	10,12	9,11	11	16,18	6,9	8,11	12,13	
103	m	72	Parietal	pt	GSC#440	CVCL_A9VT	M	25	664	X,Y	11,12	9,10	8,9	9,11	15,17	6,9	8,11	9,11	
104	f	67	Frontal	pt	GSC#441	CVCL_A9VU	UM	30	692	X	12,13	12	10,11	11	14,17	7,9.3	8,12	10	
105	f	51	Frontal	pt	GSC#442	CVCL_A9VV	UM	30	667	X	11	12	7,10	8,12	17	9.3	8	12	
106	m	54	Parietal	pt	GSC#445	CVCL_A9VW	UM	30	701	X,Y	10,12	10,11	11	9,12	17,18	7,8	8	10,11	
107	m	52	Frontal	pt	GSC#447P	CVCL_A9VX	M	25	702	X	12,13	11	11,12	11,12	17,18	6,9	8	11,12	
108	m	64	Temporal	pt	GSC#448	CVCL_A9VY	M	20	669	X,Y	11	12	8,13	11,12	16,17	9	8	10,12	
109	m	75	Temporal	pt	GSC#449	CVCL_A9VZ	M	20	703	X,Y	11	11	8,11	13, 14	15,17	8,9.3	8,12	10	
110	m	76	Temporal	pt	GSC#450	CVCL_A9W0	M	20	694	X	11,12	11,13	9,11	11,13	17,18	8,9	8	10	
111	f	67	Temporal	pt	GSC#452C	CVCL_A9W1	M	20	704	X	12,13	8,12	8	11	17	6,9.3	8	10,12	
111	f	67	Temporal	pt	GSC#452P	CVCL_A9W2	M	20	705	X	12,13	8,12	8	11	17	6,9.3	8	10,12	
112	m	77	Temporal	pt	GSC#454	CVCL_A9W3	M	25	706	X	10,11	11	11, 12	11,12	16,17	9,9.3	8,9	12	
113	m	74	Temporal	pt	GSC#455	CVCL_A9W4	UM	25	707	X,Y	12	8	11	12	16,20	6,9.3	11	10	

*Notes:* Shown are STR profiles generated in our study from GSCs. Tumor location, primary/recurrent tumor status of glioblastoma tumors that originated GSCs, MGMT gene methylation status and tumor proliferation index (Ki67%) of GSCs are also shown. Previous studies involving GSCs are indicated by the number of reference in the last column of the table.

Abbreviations: f, female; GSC, glioblastoma stem‐like cell; Ki67%, % cells positive to Ki67 proliferation antigen; m, male; M, methylated; MGMT, O6‐methylguanine DNA methyltransferase; NA, not analyzed; pt, primary tumor; rt, recurrent tumor; STR, short tandem repeat; UM, unmethylated.

For GSCs, the MSI status was not analyzed since its frequency, as determined through the amplification of the monucleotide loci, is rare in glioblastoma tumors. Single loci MSI are observed in a low percentage of glioblastoma samples and the presence of high MSI is not a typical feature of this tumor.^18^

GSCs were also analyzed for the expression of the markers CD133 and Sox2 (Table S5). Expression of CD133 (n = 45) and Sox2 (n = 43) was 25.0% ± 5.0% (mean ± SEM; range 0%‐95.9%) and 66.1% ± 4.8% (mean ± SEM; range 0.3%‐97.3%), respectively. Our data show that GSCs display different levels of CD133 and Sox2 expression, regardless of their stemness properties. The extensive intra‐ and intertumor heterogeneity of glioblastoma,^19^ may account for the inability of surface markers CD133 and Sox2 to characterize GSCs.

### 3.2 Identification of STR profiles

Eighteen CTSCs and 103 GSCs were genetically identified using STR profiling with two different kits using 10 or 16 different loci (Supplementary Material and Methods). To protect the identity of the subjects, eight core STR loci plus Amelogenin were used to report on the identity of a given sample. The following core loci were recommended, D5S818, D13S317, D7S820, D16S539, vWA, TH01, TPOX and CSF1PO.^20^, ^21^ The information about the other loci can be shared in strict confidence to researchers and biorepositories.^22^, ^23^ Tables 1 and 2 show STR profiles of CTSCs and GSCs, respectively. As an example, the STR profile for GSC#447P cell line (STR ID 702) is given in Figure S1.

Amelogenin marker was used for gender determination.^24^ This marker is located on the gonosomal chromosomes, in Xp22.1‐22.3 (AMELX) and Yp11.2 (AMELY). Its DNA fragments, obtained in PCR using specific primers for intron 3, differ by 6 bp, between X and Y chromosomes, because the AMELX contains a 6 bp deletion in intron 3 (CRCh38.12, 11,296,918 and 11,296,919, GenBank).

Failure in amelogenin sex gene detection is rare in healthy individuals^25^ and in diploid cells. However, AMELY chromosomal losses are highly frequent in tumor cell lines, hence exclusive AMELX is not predictive for the authentication in samples originated from male patients.^12^ Cell lines derived from tumor samples can lose part of the Y chromosome during culture, therefore sex determination can only be indicative.

Among the GSCs that arose from 66 male individuals, 16 cell lines showed only AMELX (GSC#1, GSC#83 and 83.2, GSC#142, GSC#148, GSC#170, GSC#206, GSC#309S, GSC#381, GSC#393, GSC#394, GSC#395, GSC#411, GSC#447P, GSC#450, GSC#454). Therefore, about 24% of the male‐derived cell line profiles seem to have lost AMELY. It has been reported that about 40% to 45% of cell lines purportedly derived from males lacked the AMELY allele.^26^

### 3.3 Comparison of STR profiles

The analysis of STR profiles of CTSCs and GSCs by CLIMA^13^ revealed that some cell lines were derived from the same individual. Among GSCs, the following cell lines derived from the same patient have profile similarity, GSC#23C and GSC#23p (93.75% of similarity); GSC#83, GSC#83.2 (93.75% of similarity); GSC#30P and GSC#30PT (100% of similarity); GSC#195 and GSC#195V (100% of similarity); GSC#314P and GSC#314C (100% of similarity). The following cell lines derived from primary and secondary surgery of the same patient show profile similarity, GSC#275 and GSC#275bis (100% of similarity); GSC#394, GSC#394bis (100% of similarity).

In some cell lines, PBMCs and the primary tumor tissues (T) were also analyzed for STR profiles. Results were as follows, GSC#298 and #298 PBMCs (100% of similarity); GSC#309s, #309T and #309 PBMCs (100% of similarity); 314T and 314 PBMCs (100%); GSC#318 and #318T (100%). All the other cell lines have unique profiles, that do not match with other profiles for a percentage higher than 90%. All unique profiles have been confirmed in CLASTR.

## 4 DISCUSSION

Human cell lines obtained from CSCs represent an invaluable model for studying the properties of tumors.^27^ These cells provide new insights into the biology of tumors and models that use the CSCs are essential tools for translational research. For example, we previously demonstrated that CSC‐enriched spheroid cultures faithfully capture important features of primary colorectal tumors in terms of both genetic landscape and drug sensitivity.^2^, ^3^, ^4^, ^5^, ^6^ In a recent study, we provided an extensive analysis of CTSC response to EGFR‐targeted therapy in vivo, leading to a deeper understanding of the molecular determinants of therapy resistance and sensitivity to combination therapies.^6^ In another study on the translational impact of the CSC model, we demonstrated that resistance of GSCs to standard treatment (ie, radiation therapy and temozolomide) relates to the clinical outcome of donor patients.^3^ In addition to demonstrating the clinical relevance of CSCs, these studies suggest how this model may guide therapeutic strategies in terms of both response predictions to current treatment and more appropriate drug selection.^6^

In the present study, we detected MSI‐H in 7 out 18 CTSC cell lines (38%) that is higher than expected in CRC. There are two possible explanations for this result. The first is that 13 (72%) tumor samples, which the CTSCs were isolated from, came from right‐sided colon cancers that harbor the MSI‐H phenotype most often.^28^ An alternative explanation is that the cultured CRC cell lines show MSI‐H more frequently than the parent tumor,^29^ suggesting that MSI‐H tumors can be more easily expanded in vitro. Most of the cell lines described in this article have been used in earlier studies^1^, ^2^, ^3^, ^4^, ^5^, ^6^, ^9^, ^10^, ^11^, ^12^, ^13^ (see Tables 1 and 2 for details).

Despite the success of using cell lines as models to advance cancer research, misidentification of cell lines is a widespread problem.^7^, ^8^, ^9^, ^12^, ^30^, ^31^ Authentication testing is an effective way to solve the problem, for this reason the disclosure of false or misidentified cell lines is the principal aim of the International Cell Line Authentication Committee (www.ICLAC.org), a voluntary, independent scientific committee, established in 2012. ICLAC produces important guidelines, such as “Guide to Human Cell Line Authentication” and “Obtaining Cell Lines from Reliable Sources.” ICLAC was established after the publication of a consensus Standard for human cell line authentication by STR profiling.^12^ Cellosaurus is a cell line knowledge resource containing information about 92 500 human cell lines and reports data about problematic (contaminated/misidentified) cell lines. Recently, a collaboration between the Cellosaurus database^32^ and the Resource Identification Initiative (https://f1000research.com/articles/4-134/v2) determined the use of an unique research resource identifier to flag each established cell line for searches and data analysis.^33^

Using STR profiling, we generated for each CSC line a unique molecular identity pattern. STR profiles from CTSCs and GSCs were compared both with cell line profiles included in CLIMA 2.1 database^13^ and with cell lines of Cellosaurus using the CLASTR search tool. Besides STR profiles, for each CTSC and GSC line, clinical data of the patients are reported such as tumor location, stage and mutations, MGMT methylation status and tumor proliferation index, MSI, expression of mismatch repair proteins (Tables 1 and 2; Tables S2 and S3) and the expression of molecular markers (Tables S4 and S5).

The cell lines used in our study will be available to researchers through Material Transfer Agreement (MTA).

## CONFLICT OF INTEREST

The authors declared no potential conflicts of interest.

## ETHICS STATEMENT

Glioblastoma tissue samples were harvested from patients undergoing craniotomy at the Institute of Neurosurgery, Università Cattolica del Sacro Cuore (UCSC), Rome, Italy. All the patients provided written informed consent according to the research proposals approved by the Ethical Committee of UCSC. Fresh human colorectal cancer tissues were obtained in accordance with the standards of the ethics committee on human experimentation of the Istituto Superiore di Sanità (authorization no. CE5ISS 09/282). All the patients provided written informed consent. Cell lines obtained from tumor stem‐like cells were de‐identified to protect patient health information.

## Supporting information


**Appendix** S1: Supporting Information

## Data Availability

The cell lines used in this study will be available to researchers through a Material Transfer Agreement (MTA). The presented STR profiles and clinical information of our CSCs are available in the Cellosaurus database (ExPASy), under the RRID numbers listed in Tables 1 and 2. In addition, the STR profiles are also uploaded to the Cell Line Integrated Molecular Authentication Database 2.1 (CLIMA 2.1) (http://bioinformatics.hsanmartino.it/clima2/). Other data supporting the findings of this study are available from the corresponding author upon request.
