# Supplementary material for: Short tandem repeat profiling for the authentication of cancer stem‐like cells
Source: Int J Cancer. 2020 Nov 9;148(6):1489–98. doi: 10.1002/ijc.33370 (PMC7894552; doi:10.1002/ijc.33370)
Supplement: Supplementary file 1 — Appendix S1: Supporting Information [file IJC-148-1489-s001.pdf]

STR profiling for the authentication of cancer stem-like cells

Paola Visconti, Federica Parodi, Barbara Parodi, Lucia Casarino, Paolo Romano, Mariachiara Buccarelli, Roberto Pallini, Quintino Giorgio D'Alessandris, Andrea Montori, Emanuela Pilozzi, Lucia Ricci-Vitiani.

Supplementary Materials and Methods

Table 1S: CLIMA 2.1 database datasets.

Table 2S: Genetic alterations of tumor-specific genes in CTSCs: gene name, protein change, coding nucleotide number and mutation type.

Table 3S: Stability status of the mononucleotide repeats BAT-25, BAT-26, NR-21, NR-24, MONO-27 in CTSCs; expression of 4 mismatch repair proteins MLH1, MSH2, MSH6 and PMS2 investigated in tumor tissues corresponding to CTSC lines that showed MSI-H.

Table 4S: Expression of the surface markers CD133 and Ber-Ep4 in CTSCs evaluated by flow cytometry and showed as percentage of positive tumor cells.

Table 5S: Expression of the surface markers CD133 and SOX2 in a panel of GSC lines evaluated by flow cytometry and showed as percentage of positive tumor cells.

Figure 1S: Shown is the STR profile for GSC#447P cell line (STR ID 702) generated using the GeneMapper ID® software, Version 3.2, using the the PowerPlex® 16 HS System (Promega). The Y-axis shows relative peak heights for the PCR products that are generated. On the X-axis the PCR length are shown. The gray columns indicate the length of the known PCR products within a locus. All locus names are shown in gray quadrants. The allele calls and the heights of the peaks are indicated in the boxes below the peaks along the X axes.

Supplementary Materials and Methods

CSCs cultures

GSC cultures were established from tumor specimens through mechanical dissociation and culturing in DMEM/F12 serum-free medium containing 2 mM glutamine, 0.6% glucose, 9.6 g/ml putrescine, 6.3 ng/ml progesterone, 5.2 ng/ml sodium selenite, 0.025 mg/ml insulin, and 0.1 mg/ml transferrin sodium salt (Sigma-Aldrich, St. Louis, MO), supplemented with EGF and bFGF. Under these conditions, tumor cells grow as spheroid clusters expressing stem-cell markers, such as CD133, Sox2, Musashi, and nestin. CD133 expression of GSCs was detected by AC133-PE antibody or PE-conjugated mouse IgG1 isotype control antibody (Miltenyi Biotec, Bologna, Italy). The expression of Sox2 was analyzed by PerCP-Cy^{TM} 5.5 Mouse anti-Sox2 or PerCP-Cy^{TM} 5.5 mouse IgG1 isotype Control (BD, Becton Dickinson and Company, Milan, Italy). Viable cells were identified using 7-amino actinomycin D (7AAD; Sigma Aldrich, St. Louis, MO). Cells were analyzed with FACSCanto flow cytometer (Becton Dickinson). The MGMT promoter methylation of GSCs was assessed after DNA extraction from cell pellets with Wizard genomic DNA purification kit (Promega, Milan, Italy).[14]

Apart from sphere generation *in vitro*, the stem-like phenotype of GSCs was assessed by self-renew capacity, co-expression of astrocytic and neuronal markers, and generation of xenografts histologically mimicking the parent tumor.

To obtain colon cancer stem cell culture, surgical specimens were washed several times with phosphate buffered saline (PBS) and were incubated overnight at 4°C in DMEM-F12 containing 25 units/ml of penicillin, 25 mg/ml streptomycin and 10 mg/ml amphotericin B. The day after, samples are subjected to mechanical dissociation followed by enzymatical dissociation in DMEM containing collagenase II (1,5 mg/ml) and DNase I (20 μgr/ml) for about 10-20 min (depending on samples size). At the end of the enzymatic digestion, cell suspension is filtered by using a 100 μm nylon cell strainer (Falcon). The resulting cancer cells were cultured in stem cell medium in the presence of 20 ng/ml of human recombinant epidermal growth factor and 10 ng/ml of human recombinant basic fibroblast growth factor (the same used for GSCs).
Colorectal tumor samples were obtained from Department of Clinical and Molecular Medicine, Sant'Andrea Hospital, University La Sapienza, Rome, Italy. Glioblastoma samples were obtained from Institute of Neurosurgery, Fondazione Policlinico Universitario A. Gemelli IRCCS - Università Cattolica del Sacro Cuore, Rome, Italy.

Molecular analyses in CTSCs and colorectal tumors

Microsatellite instability (MSI) detection was performed on mononucleotide repeats BAT-25, BAT-26, NR-21, NR-24, MONO-27 and pentanucleotide repeats Penta C and Penta D for sample identification. The amplification conditions were carried out following manufacturer's instructions. PCR products were denatured in deionized formamide with Internal Lane Standard 600 (Promega) for allele sizing and analyzed on a 3130xl Genetic Analyzer using GeneMapper 4.0 Software (Applied Biosystems). DNA extracted from normal mucosa of the corresponding CTSC generated

line was amplified. Allelic sizes from matching normal and CTSC samples were compared and considered MSI unstable if there was a shift of 3bp or more in the CTSC allele. Samples were classified as MSI-High (MSI-H): - two or more markers out of a panel of five unstable, MSI-Low (MSI-L): - one out of five markers unstable and microsatellite stable (MSS): - no unstable markers. Mismatch repair protein expression was assessed by immunohistochemistry, according to the CAP protocol. Immunohistochemical staining procedure was automatically conducted through DAKO Omnis device using EnVisionFLEX IHC kit. Mismatch repair protein expression was assessed by immunohistochemistry with following antibodies: MSH2 (Monoclonal Mouse, RTU, clone FE11, Dako, AB_2631353), MSH6 (Monoclonal Rabbit, RTU, clone EP49, Dako), MLH1 (Monoclonal Mouse, RTU, clone ES05, Dako, AB_2631352), and PMS2 (Monoclonal Rabbit, RTU, clone EP51, Dako). Tumor representative blocks were selected for analysis with normal-tumor junction in order to assess staining result properly. Sections with a thickness of 2-4 μm were cut and mounted on glass slides. Adjacent normal colonic epithelium, lymphocytes, and stromal cells served as positive internal controls.

The expression of the stem cell marker CD133 and the epithelial antigen Ber-EP4 in CTSCs have been evaluated by flow cytometry. The following antibodies were used: anti-CD133-phycoerythrin (clone AC133/1, mouse IgG1, MiltenyiBiotec Inc., Bergisch Gladbach, Germany) (AB_244342), anti-Epithelial Antigen-fluorescein isothiocyanate (clone Ber-Ep4, mouse IgG1, DakoCytomation, Denmark) or isotype-matched control antibodies (AB_871707 and AB_871706, respectively). Samples were analyzed with FACSCanto flow cytometer (Becton Dickinson, San Jose, CA) and data were analyzed with FACS Diva software (Becton Dickinson).

Molecular analyses in GSCs and glioblastoma tumors

MGMT promoter methylation patterns were studied by methylation-specific PCR using primers specific for methylated and unmethylated DNA on genomic DNA extracted from paraffin embedded tissue using QIAamp DNA mini kit (QIAGEN, Hilden, Germany). The annealing temperature was 60°C. The expression of CD133 and Sox2 in GSCs have been evaluated by flow cytometry, using the following antibodies: anti-CD133-phycoerythrin (clone AC133/1, mouse IgG1, MiltenyiBiotec Inc.) (AB_244342), anti-Sox2-PerCP-Cy 5.5 (mouse IgG1, Becton Dickinson) (AB_10646039) or isotype-matched control antibodies (AB_871707 and AB_393885, respectively). Cells were analyzed with a FACSCanto flow cytometer (Becton Dickinson) and data were analyzed with FACS Diva software (Becton Dickinson).

STR profiling

The profiling of STR sequences was performed using two different kits (Promega). STR profiling was assessed within the first 5 passages, when cell lines were frozen down as stocks in large batches, so the profile represents any sample that would be shared with other researchers. An allelic ladder was used as reference to assign the genotype and each amplification also contains

positive and negative controls. One microliter of amplified DNA was combined with loading solution mix containing Internal Lane Standard 600 (ILS600) and Hi-Di formamide. Samples were heat denatured at 95°C for 3 min, refrigerated on ice for at least 3 min, transferred onto a plate and placed in the sequencer ABI Prism® 3100 Genetic Analyzer (Applied Biosystems) with a separation matrix for performing DNA sequencing and fragment analysis. The ABI PRISM® 3100 Genetic Analyzer is a 16–capillary electrophoresis system allowing both sequencing and fragment analysis to be performed at medium-to-high throughput, using a 36 cm array filled with POP-7™ polymer. STR profiles were analyzed by the GeneMapper ID®software (Applied Biosystems), Version 3.2. The profiling of STR sequences was performed using two different kits: initially samples (from STR ID 291 to 510) were analyzed using the GenePrint® 10 System (Promega, Fitchburg, WI, USA), that co-amplifies in a single polymerase chain reaction (PCR) 10 loci: D21S11, TH01, TPOX, vWA, CSF1PO, D16S539, D13S317, D5S818, D7S820, and amelogenin. Subsequently samples (from STR ID 579 to 775 and 251, 413, 416, 418, 425, 427, 428, 446, 500) were profiled using the PowerPlex® 16 HS System (Promega), which co-amplifies 16 loci: D18S51, D21S11, TH01, D3S1358, FGA, TPOX, D8S1179, vWA, CSF1PO, D16S539, D7S820, D13S317, D5S818, Amelogenin, Penta E and Penta D. Information on main kits for STR profiling and descriptions of related loci are available in STRBase (hiips://strbase.nist.gov/).

To compare profiles in CLIMA database the ratio between the number of matches and the number of distinct values in the matched profile in the database (Standard Percent Match, StPM) is computed, which is a modified version of the Masters algorithm ((hiip://bioinformatics. hsanmartino.it/clima2/). All profiles of cell lines matching the query with an StPM over the specified threshold are returned, sorted by descending StPM.

Table 1S: CLIMA 2.1 database datasets.

Dataset	Name	Description	Platform	N° of cell lines
1	PNAS	Results obtained from leading cell banks and cancer research institutes. Masters et al. 2001, PMID 11416159	Platform 1, silica-gel-based purification kit (SGM) (Qiagen, Crawley, UK)	223 cell lines
2	ATCC	STR profiles made available by ATCC	Platform 2, Promega PowerPlex® 1.2 system	1,097 cell lines
3	JBRC	STR profiles made available by the JBRC	Platform 2	828 cell lines
4	ICLC1	STR profiles obtained from Cell Bank Interlab Cell Line Collection (ICLC)	Platform 2	15 cell lines
5	ICLC2	ICLC	Platform 2	170 cell lines
6	COG	STR profiles made available by Children Cancer Repository, a Children's Oncology Group Resource Laboratory (COG)	Platform 2	1,186 cell lines
7	DSMZ	STR profiles made available by DSMZ biobank	Platform 2	1,920 cell lines
8	ICLC3	STR profiles obtained at ICLC from CTSCs and GSCs described in this study and other cell lines	Platform 2	148 cell lines

Table 1S: Shown are the eight datasets included in CLIMA 2.1 database. For each dataset the following information is available: name, description, platform and N° of cell lines.

ATCC: American Type Culture Collection; COG: Children's Oncology Group; DSMZ: Deutsche Sammlung von Mikroorganismen und Zellkulturen ; ICLC: Cell Bank Interlab Cell Line Collection; JBRC: Japanese Collection of Research Bioresources Cell Bank; PNAS: Proceedings of the National Academy of Sciences

Table 2S: Genetic alterations of tumor-specific genes in CTSCs: gene name, protein change, coding nucleotide number and mutation type

**CTSC#1.1**	
NRAS	p.Gly12Asp, c.35G>A (snv)
PIK3CA	p.Glu542Lys, c.1624G>A (snv)
APC	p.Thr182IlefsTer2, c.540_543delACAA (deletion); p.Arg259Trp, c.775C>T (snv); p.Glu1284Ter, c.3850G>T (snv); p.Glu1317Gln, c.3949G>C (snv)
TCF7L2	p.Leu200ProfsTer10, c.593_594insC (insertion)
SMAD4	p.Arg361Cys, c.1081C>T (snv)

**CTSC#1.2**	
NRAS	p.Gly12Asp, c.35G>A (snv)
CTNNB1	p.Pro606Ala, c.1816C>G (snv)
PIK3CA	p.Glu542Lys, c.1624G>A (snv)
APC	p.Thr182IlefsTer2, c.540_543delACAA (deletion); p.Arg259Trp, c.775C>T (snv); p.Glu1284Ter, c.3850G>T (snv); p.Glu1317Gln, c.3949G>C (snv)
TCF7L2	p.Leu200ProfsTer10, c.593_594insC (insertion)
SMAD4	p.Arg361Cys, c.1081C>T (snv)

**CTSC#18**	
KIAA1804	p.Arg345GlyfsTer12, c.1033delC (deletion)
FBXW7	p.Lys647Glu, c.1939A>G (snv)

APC	p.Ala199Val, c.596C>T (snv); p.Gly2250Asp, c.6749G>A (snv)
PTEN	p.Asn323MetfsTer21, c.963delA (deletion)
TCF7L2	p.Ser569ProfsTer33, c.1700delC (deletion)
KRAS	p.Gly12Val, c.35G>T (snv)
ACVR1B	p.Trp501Ter, c.1503G>A (snv)
TP53	p.Asp148Ter, c.441dupT (insertion)
SOX9	p.Val80Glu, c.239T>A (snv); p.Tyr84Cys, c.251A>G (snv); p.Lys166del, c.496_498delAAG (deletion)

**CTSC#85**	
APC	p.Arg283Ter, c.847C>T (snv); p.Ser1415ArgfsTer4, c.4245delT (deletion)
PTEN	p.Val54Ala, c.161T>C (snv)
KRAS	p.Ala146Thr, c.436G>A (snv)
SMAD4	p.Arg361Cys, c.1081C>T (snv)

**CTSC#383**	
CTNNB1	p.Trp690Arg, c.2068T>C (snv)
APC	p.Arg564Ter, c.1690C>T (snv); p.Ala612HisfsTer18, c.1834delG (deletion)
KRAS	p.Ala146Thr, c.436G>A (snv); p.Asp54Tyr, c.160G>T (snv)
TP53	p.Ser215Asn, c.644G>A (snv)
SMAD2	p.Glu326Lys, c.976G>A (snv)

CTSC#389

PIK3CA p.His1047Arg, c.3140A>G (snv)

FBXW7 p.Ala626Val, c.1877C>T (snv)

KRAS p.Gly12Val, c.35G>T (snv)

TP53 p.Arg273Cys, c.817C>T (snv)

SOX9 p.Gly461Cys, c.1381G>T (snv)

SMAD4 p.Gln366Ter, c.1096C>T (snv); p.Arg497His, c.1490G>A (snv)

CTSC#393

CTNNB1 p.Val325Ile, c.973G>A (snv)

PIK3CA p.Glu453Lys, c.1357G>A (snv); p.Glu970Val, c.2909A>T (snv)

FBXW7 p.Gln277Ter, c.829C>T (snv); p.Glu110Lys, c.328G>A (snv)

APC p.Ala440Thr, c.1318G>A (snv); p.Ala759AsnfsTer2, c.2273_2285delAAGCCCTAGAAGC (deletion)

CTSC#398

PIK3CA p.Arg524Lys, c.1571G>A (snv)

APC p.Met314Ile, c.942G>T (snv); p.Glu991Ter, c.2971G>T (snv)

KRAS p.Gln61His, c.183A>T (snv)

TP53 p.Arg196Ter, c.586C>T (snv)

SMAD4 p.Arg361Cys, c.1081C>T (snv)

CTSC#416

NRAS p.Leu133Gln, c.398T>A (snv); p.Phe90Leu, c.270T>A (snv); p.Arg41Ser, c.123A>T (snv); p.Ile36Val, c.106A>G (snv)

KIAA1804 p.Val582Ile, c.1744G>A (snv); p.Gln970Leu, c.2909A>T (snv)

CTNNB1 p.Gly48Val, c.143G>T (snv); p.Gln130Arg, c.389A>G (snv); p.Cys381Phe, c.1142G>T (snv); p.Ala467Thr, c.1399G>A (snv); p.Glu705Lys, c.2113G>A (snv); p.Val750Ala, c.2249T>C (snv)

PIK3CA p.Arg38Cys, c.112C>T (snv); p.Glu85Lys, c.253G>A (snv); p.Leu153His, c.458T>A (snv); p.Gln374Ter, c.1120C>T (snv); p.Leu445Pro, c.1334T>C (snv); p.Gly451Glu, c.1352G>A (snv); p.Tyr508Phe, c.1523A>T (snv); p.Glu674Gly, c.2021A>G (snv); p.Lys700Glu, c.2098A>G (snv)

APC p.Val47Ile, c.139G>A (snv); p.Arg374Gln, c.1121G>A (snv); p.Gln532Arg, c.1595A>G (snv); p.Arg805Ter, c.2413C>T (snv); p.Gly877Asp, c.2630G>A (snv); p.Arg1103Gly, c.3307A>G (snv); p.Glu1464ValfsTer8, c.4385_4388delAGAG (deletion); p.Lys1762Glu, c.5284A>G (snv); p.Val1834Ile, c.5500G>A (snv); p.Leu1865Ile, c.5593C>A (snv); p.Thr2178Ser, c.6532A>T (snv); p.Arg2439Cys, c.7315C>T (snv); p.Ser2799Pro, c.8395T>C (snv)

BRAF p.Ala561Thr, c.1681G>A (snv); p.Gly518Ser, c.1552G>A (snv); p.Lys507Arg, c.1520A>G (snv)

PTEN p.Tyr27His, c.79T>C (snv); p.Lys267ArgfsTer9, c.795delA (deletion); p.Asn323MetfsTer21, c.963delA (deletion); p.Asn334Asp, c.1000A>G (snv);

TCF7L2 p.Thr135Ser, c.403A>T (snv); p.Pro221Leu, c.662C>T (snv); p.Ile253Ser, c.758T>G (snv); p.Ala419Thr, c.1255G>A (snv); p.Asp432ThrfsTer59, c.1292delG (deletion); p.Pro435Ser, c.1303C>T (snv)

ACVR1B p.Leu140Phe, c.418C>T (snv); p.Phe332Ser, c.995T>C (snv); p.Ala398LeufsTer36, c.1191delG (deletion)

TP53 p.Leu344Pro, c.1031T>C (snv); p.Arg267Gln, c.800G>A (snv); p.Met1?, c.3G>T (snv)

MAP2K4 p.Glu203Lys, c.607G>A (snv); p.Pro326Thr, c.976C>A (snv); p.Glu360Lys, c.1078G>A (snv)

SOX9 p.Ala481Val, c.1442C>T (snv)

SMAD2 p.Arg462His, c.1385G>A (snv)

SMAD4 p.Lys45Glu, c.133A>G (snv); p.Thr174Ala, c.520A>G (snv); p.Gln410Arg, c.1229A>G (snv); p.Ser483Asn, c.1448G>A (snv); p.Leu529Ter, c.1586T>A (snv);

AMER1 p.Leu869Pro, c.2606T>C (snv); p.Arg497Ter, c.1489C>T (snv); p.Val442Ile, c.1324G>A (snv); p.Ala333Glu, c.998C>A (snv)

CTSC#417

PIK3CA p.Arg524Lys, c.1571G>A (snv)

APC p.Met438Thr, c.1313T>C (snv); p.Arg876Ter, c.2626C>T (snv)

TP53 p.Arg282Trp, c.844C>T (snv)

CTSC#430

KIAA1804 p.Glu655Val, c.1964A>T (snv)

PIK3CA p.Gly248Ser, c.742G>A (snv); p.Glu563Lys, c.1687G>A (snv)

FBXW7 p.Arg179Cys, c.535C>T (snv); p.His104Tyr, c.310C>T (snv)

APC p.Gly97Arg, c.289G>A (snv); p.Ser1465TrpfsTer3, c.4385_4386delAG (deletion)

PTEN p.Leu57TrpfsTer42, c.166delT (deletion); p.Lys267ArgfsTer9, c.795delA (deletion)

SMAD4 p.Thr34Lys, c.101C>A (snv); p.His287Gln, c.861T>G (snv)

CTSC#432

CTNNB1 p.Arg486His, c.1457G>A (snv)

TCF7L2 p.Pro300Leu, c.899C>T (snv)

KRAS	p.Ala146Thr, c.436G>A (snv)
TP53	p.Val272Leu, c.814G>T (snv)

**CTSC#438**	
PIK3CA	p.Leu443Ser, c.1328T>C (snv); p.Glu545Lys, c.1633G>A (snv)
BRAF	p.Val600Glu, c.1799T>A (snv)
SOX9	p.Thr243ProfsTer10, c.724delA (deletion)
SMAD2	p.Arg182Ter, c.544C>T (snv)
AMER1	p.Arg699His, c.2096G>A (snv)

**CTSC#446**	
NRAS	p.Cys186Tyr, c.557G>A (snv)
KIAA1804	p.Phe321Ser, c.962T>C (snv); p.Pro491Leu, c.1472C>T (snv); p.Leu507His, c.1520T>A (snv); p.Ser614Pro, c.1840T>C (snv); p.Pro982Leu, c.2945C>T (snv); p.Ser1036Phe, c.3107C>T (snv)
CTNNB1	p.Asp412Gly, c.1235A>G (snv); p.Arg717His, c.2150G>A (snv)
PIK3CA	p.Tyr182Cys, c.545A>G (snv); p.Cys378Trp, c.1134T>G (snv); p.Cys407Tyr, c.1220G>A (snv); p.Lys548Asn, c.1644A>T (snv); p.Leu719Phe, c.2155C>T (snv); p.Gln721Leu, c.2162A>T (snv); p.Phe897Val, c.2689T>G (snv); p.His931Tyr, c.2791C>T (snv); p.Gln1014Ter, c.3040C>T (snv); p.Ile1022Val, c.3064A>G (snv)
FBXW7	p.Ser678Ter, c.2033C>G (snv); p.Ser142Arg, c.424A>C (snv)
APC	p.Tyr96MetfsTer29, c.285delT (deletion); p.Gln264Leu, c.791A>T (snv); p.Gly400Val, c.1199G>T (snv); p.Trp421Ter, c.1262G>A (snv); p.Thr675Ile, c.2024C>T (snv); p.Asn1161Asp, c.3481A>G (snv); p.Lys1350Arg, c.4049A>G (snv); p.Phe1354Ser, c.4061T>C (snv); p.Gln1367Ter, c.4099C>T (snv); p.Asp1532Glu, c.4596C>G (snv); p.Asp1558Asn, c.4672G>A

	(snv); p.Leu1601Ter, c.4802T>A (snv); p.Phe1933Ile, c.5797T>A (snv); p.Gln2376Ter, c.7126C>T (snv); p.Lys2445Ile, c.7334A>T (snv); p.Pro2540Ser, c.7618C>T (snv); p.Ser2555Pro, c.7663T>C (snv); p.Leu2718Phe, c.8154G>T (snv); p.Gln2727ArgfsTer12, c.8177delT (deletion)
PTEN	p.His93Arg, c.278A>G (snv); p.Leu182Ser, c.545T>C (snv)
TCF7L2	p.Gln263Arg, c.788A>G (snv)
KRAS	p.Asp105Val, c.314A>T (snv)
ACVR1B	p.Met361Leu, c.1081A>C (snv); p.Glu482Gly, c.1445A>G (snv)
TP53	p.Phe270Cys, c.809T>G (snv); p.Ala189ProfsTer58, c.565delG (deletion)
MAP2K4	p.Pro71Leu, c.212C>T (snv); p.Tyr113Phe, c.338A>T (snv); p.Gln327Arg, c.980A>G (snv)
SOX9	p.Pro108His, c.323C>A (snv)
SMAD2	p.Leu254Phe, c.760C>T (snv); p.Asn34Asp, c.100A>G (snv)
SMAD4	p.His67Arg, c.200A>G (snv); p.Thr73Ala, c.217A>G (snv); p. Thr77Ala, c.229A>G (snv); p.Ser150Cys, c.448A>T (snv); p.Pro431Leu, c.1292C>T (snv); c.1658G>A (p.=) (snv)
AMER1	p.Glu1009Gly, c.3026A>G (snv); p.Ala962LeufsTer14, c.2884delG (deletion); p.His910Tyr, c.2728C>T (snv); p.Lys87Arg, c.260A>G (snv)

**CTSC#482**	
KIAA1804	p.Cys190Phe, c.569G>T (snv); p.Gln192SerfsTer53, c.573delG (deletion)
PIK3CA	p.Pro3Thr, c.7C>A (snv); p.His1047Arg, c.3140A>G (snv)
FBXW7	p.Arg465His, c.1394G>A (snv)
APC	p.Asn436IlefsTer18, c.3103delA (deletion); p.Ser1465TrpfsTer3, c.4385_4386delA>C (deletion); p.Asp1636MetfsTer14, c.4902delG (deletion)

BRAF	p.Val600Glu, c.1799T>A (snv)
TCF7L2	p.Glu65Ter, c.193G>T (snv); p.Ser79Phe, c.236C>T (snv); p.Ser82Ile, c.245G>T (snv); p.Arg89Ser, c.267G>T (snv); p.Ile106Phe, c.316A>T (snv); p.Asn118ThrfsTer37, c.348delC (deletion); p.Asp486Tyr, c.1456G>T (snv); p.Gln590SerfsTer12, c.1766delC (deletion)
TP53	p.Arg213Gln, c.638G>A (snv); p.Leu201Ter, c.602T>A (snv)
SOX9	p.Gln368Lys, c.1102C>A (snv); p.Leu382Gln, c.1145T>A (snv)
AMER1	p.Glu920Ter, c.2758G>T (snv)

**CTSC#510**	
KIAA1804	p.Ala176Pro, c.526G>C (snv); p.Asn182Tyr, c.544A>T (snv)
CTNNB1	p.Thr41Ile, c.122C>T (snv)
APC	p.Gln1752Ter, c.5254C>T (snv)
TCF7L2	p.Asp91Val, c.272A>T (snv); p.Met411Thr, c.1232T>C (snv)
KRAS	p.Gln61Lys, c.181C>A (snv)
TP53	p.Ter394AspfsTer28, c.1180_1182insGAC (insertion); p.Ser392Ter, c.1175C>A (snv); p.Glu388Lys, c.1162G>A (snv)
SOX9	p.Met10Ile, c.30G>T (snv); p.Gln175Ter, c.523C>T (snv); p.Ser279ArgfsTer104, c.837delC (deletion); p.Pro299Gln, c.896C>A (snv); p.His380Asn, c.1138C>A (snv); p.Phe423AlafsTer156, c.1265_1266insGGCT (insertion)
SMAD4	p.Asn107MetfsTer3, c.316delA (deletion); p.Arg361His, c.1082G>A (snv)
AMER1	p.Pro1058Leu, c.3173C>T (snv); p.Arg531Gln, c.1592G>A (snv); p.Gly28GlufsTer25, c.81delA (deletion)

Legend: snv= single nucleotide variation

Table 2S. Shown are genetic alterations of tumor-specific genes in CTSCs. Mutations in the following genes were analyzed: *ACVR1B, AMER1, APC, BRAF, CTNNB1, FBXW7, KIAA1804, KRAS, MAP2K4, NRAS, PIK3CA, PTEN, SMAD2, SMAD4, SOX9, TCF7L2, TP53*. Mutations are described as protein change, coding nucleotide number and mutation type.

		Detection results in mononucleotide loci						Detection results in IHC assays for MMR proteins			
**Cell Line Name**	**MSI Status**	BAT-25	BAT-26	NR-21	NR-24	MONO-27		MLH1	PMS2	MSH2	MSH6
**CTST#CRO**	MSS	-	-	-	-	-					
**CTSC#1.1**	MSS	-	-	-	-	-					
**CTSC#1.2**	MSS	-	-	-	-	-					
**CTSC#18**	MSI-H	+	+	+	+	+		-	-	+	+
**CTSC#85**	MSS	-	-	-	-	-					
**CTSC#383**	MSS	-	-	-	-	-					
**CTSC#389**	MSI-H	+	+	+	+	+		+	-	+	+
**CTSC#393**	MSS	-	-	-	-	-					
**CTSC#398**	MSS	-	-	-	-	-					
**CTSC#417**	MSI-L	+	-	-	-	-					
**CTSC#416**	MSI-H	+	+	+	+	+		+	+	-	-
**CTSC#430**	MSI-H	+	+	+	+	+		-	-	+	+
**CTSC#432**	MSS	-	-	-	-	-					
**CTSC#438**	MSI-H	+	+	+	+	+		-	-	+	+
**CTSC#482**	MSI-H	+	+	+	+	+		-	-	+	+
**CTSC#446**	MSS	-	-	-	-	-					
**CTSC#510**	MSI-H	+	+	+	+	+		-	-	+	+
**CTSC#553**	MSS	-	-	-	-	-					

Mononucleotide loci key interpretation:
(+) unstable marker
(-) stable marker
IHC detection key interpretation:
(+) positive nuclear staining ("no loss of expression")
(-) no nuclear staining ("loss of expression")

Table 3S: Shown in the left side of the table is the stability status of the mononucleotide repeats BAT-25, BAT-26, NR-21, NR-24, MONO-27 in CTSCs.
MSI-H : MSI-High , MSI-L : MSI-Low, MSS: microsatellite stable.
Shown in the right side of the table is the expression of 4 mismatch repair proteins MLH1, MSH2, MSH6, and PMS2, investigated in tumor tissues corresponding to CTSC lines that showed MSI-H.

Cell line name	PATIENTS	CD133 (%)	Ber-Ep4 (%)
	CASE#		
**CTSC#1.1**	**1.1**	89.4	99.3
**CTSC#1.2**	**1.2**	92.0	98.6
**CTSC#18**	**18**	47.6	83.0
**CTSC#CRO**	**CRO**	88.3	96.0
**CTSC#85**	**85**	37.7	89.0
**CTSC#383**	**383**	84.0	98.1
**CTSC#389**	**389**	49.0	82.8
**CTSC#393**	**393**	85.3	98.8
**CTSC#398**	**398**	81.1	86.5
**CTSC#416**	**416**	28.2	97.1
**CTSC#417**	**417**	23.2	93.4
**CTSC#430**	**430**	98.1	99.8
**CTSC#432**	**432**	24.7	96.3
**CTSC#446**	**446**	66.7	100
**CTSC#510**	**510**	62.4	94.6
**CTSC#438**	**438**	31.3	95.9
**CTSC#482**	**482**	52.3	97.2
**CTSC#553**	**553**	99.3	99.3

Table 4S: Expression of the surface markers CD133 and Ber-Ep4 in CTSCs evaluated by flow cytometry and showed as percentage of positive tumor cells.

Cell line name	Tumor	%CD133	%SOX2
	CASE#		
GSC#1	BTSC#1	95.8	95.2
GSC#10	BTSC#10	23	94.9
GSC#23C	BTSC#23C	14.2	97.3
GSC#23P	BTSC#23P	14.3	89.2
GSC#30P	BTSC#30P (C)	0.2	47.4
GSC#30pt	BTSC#30PT (P)	0	47.4
GSC#61	BTSC#61	2.5	81.6
GSC#62	BTSC#62	82.7	92.9
GSC#67	BTSC#67	0.2	80
GSC#68	BTSC#68	1.2	90.9
GSC#70	BTSC#70	17.3	38
GSC#74	BTSC#74	1.5	30.95
GSC#76	BTSC#76	1.8	88.4
GSC#83	BTSC#83 (P)	0.2	77.7
GSC#83.2	BTSC#83.2 (C)	1.7	77.7
GSC#112	BTSC#112	61.5	77.2
GSC#120	BTSC#120	83.8	84
GSC#147	BTSC#147	0.8	80.3
GSC#148	BTSC#148	94.8	94.3
GSC#151	BTSC#151	0.25	6.2
GSC#163	BTSC#163	1.2	89.2
GSC#169	BTSC#169	6.6	47.4
GSC#170	BTSC#170	0	2.4
GSC#171	BTSC#171	3.3	95.1
GSC#172	BTSC#172	2.7	94.8
GSC#181	BTSC#181	33.5	72.1
GSC#184	BTSC#184	18.6	na
GSC#188	BTSC#188	1.6	18.5
GSC#191	BTSC#191	29.8	83.7
GSC#195	BTSC#195	0	20.7
GSC#195V	BTSC#195v	0.4	20.7
GSC#196	BTSC#196	88.3	0.3
GSC#204	BTSC#204	84.9	55.2
GSC#206	BTSC#206	75.8	82.7
GSC#208	BTSC#208	15.9	2
GSC#209	BTSC#209	0.3	91.4
GSC#210	BTSC#210	95.9	90.4
GSC#213	BTSC#213	56.3	92
GSC#220C	BTSC#220C	16.8	79.6
GSC#221	BTSC#221	64.3	91.1
GSC#242	BTSC#242	0.2	9.25
GSC#257	BTSC#257	6.2	75
GSC#262	BTSC#262	10.1	81.2
GSC#275	BTSC#275	11	76.6
GSC#277	BTSC#277	3	na

Table 5S: Expression of the surface markers CD133 and SOX2 in a panel of GSC lines evaluated by flow cytometry and showed as percentage of positive tumor cells.

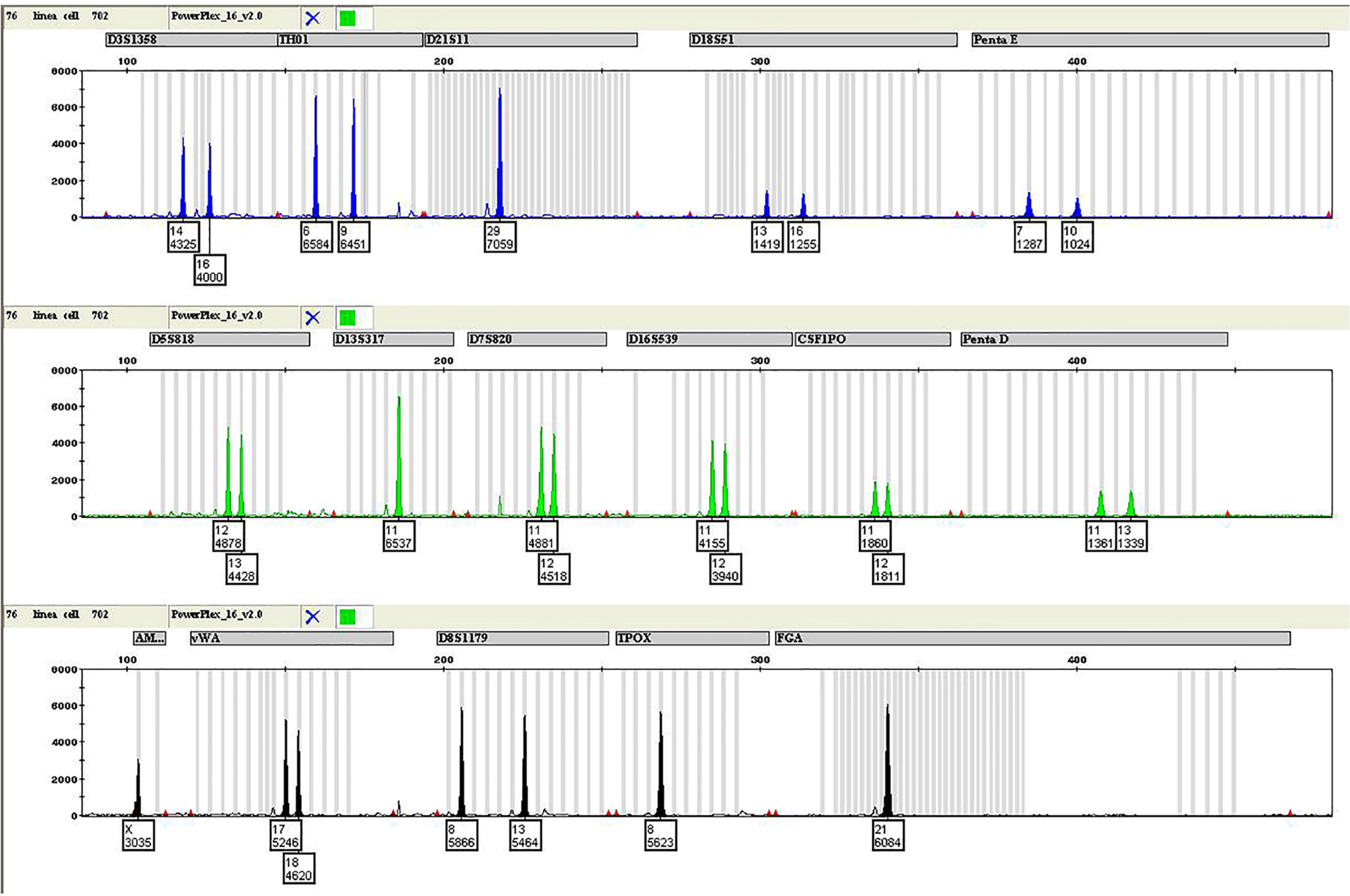
76 linea cell 702
PowerPlex_16_v2.0
D3S1358
TH01
D21S11
D18S51
Penta E
14 4325
16 4000
6 6584
9 6451
29 7059
13 1419
16 1255
7 1287
10 1024
D5S818
D13S317
D7S820
D16S539
CSF1PO
Penta D
12 4878
13 4428
11 6537
11 4881
12 4518
11 4155
12 3940
11 1860
12 1811
11 1361
13 1339
AM...
vWA
D8S1179
TPOX
FGA
X 3035
17 5246
18 4620
8 5866
13 5464
8 5623
21 6084
